# CDDO-Me Protects Normal Lung and Breast Epithelial Cells but Not Cancer Cells from Radiation

**DOI:** 10.1371/journal.pone.0115600

**Published:** 2014-12-23

**Authors:** Mariam El-Ashmawy, Oliver Delgado, Agnelio Cardentey, Woodring E. Wright, Jerry W. Shay

**Affiliations:** 1 Department of Cell Biology, UT Southwestern Medical Center at Dallas, Dallas, Texas, United States of America; 2 Department of Clinical Cancer Prevention, The University of Texas MD Anderson Cancer Center, Houston, Texas, United States of America; 3 Center for Excellence in Genomics Medicine Research, King Abdulaziz University, Jeddah, Saudi Arabia; Faculty of Biochemistry, Poland

## Abstract

Although radiation therapy is commonly used for treatment for many human diseases including cancer, ionizing radiation produces reactive oxygen species that can damage both cancer and healthy cells. Synthetic triterpenoids, including CDDO-Me, act as anti-inflammatory and antioxidant modulators primarily by inducing the transcription factor Nrf2 to activate downstream genes containing antioxidant response elements (AREs). In the present series of experiments, we determined if CDDO-Me can be used as a radioprotector in normal non-cancerous human lung and breast epithelial cells, in comparison to lung and breast cancer cell lines. A panel of normal non-cancerous, partially cancer progressed, and cancer cell lines from both lung and breast tissue was exposed to gamma radiation with and without pre-treatment with CDDO-Me. CDDO-Me was an effective radioprotector when given ∼18 hours before radiation in epithelial cells (average dose modifying factor (DMF) = 1.3), and Nrf2 function was necessary for CDDO-Me to exert these radioprotective effects. CDDO-Me did not protect cancer lines tested from radiation-induced cytotoxicity, nor did it protect experimentally transformed human bronchial epithelial cells (HBECs) with progressive oncogenic manipulations. CDDO-Me also protected human lymphocytes against radiation-induced DNA damage. A therapeutic window exists in which CDDO-Me protects normal cells from radiation by activating the Nrf2 pathway, but does not protect experimentally transformed or cancer cell lines. This suggests that use of this oral available, non-toxic class of drug can protect non-cancerous healthy cells during radiotherapy, resulting in better outcomes and less toxicity for patients.

## Introduction

Although radiation therapy is a common treatment for cancer patients, ionizing radiation (IR) produces reactive oxygen species (ROS) and is known to damage cellular components in healthy cells, leading to damaged bases and DNA breaks, resulting in chromosomal aberrations, mutagenesis, carcinogenesis, and cell death [1], [2]. Not only are these effects responsible for causing radiation sickness and other toxic side effects in cancer patients treated with ionizing or proton radiation therapy, they are a particularly important consideration for first responders to nuclear accidents, astronauts on long-term space missions, or any other situation where individuals are exposed to radiation. Radiation exposure has been specifically linked to secondary cancers later in life [3]–[5].

**Figure 1:**
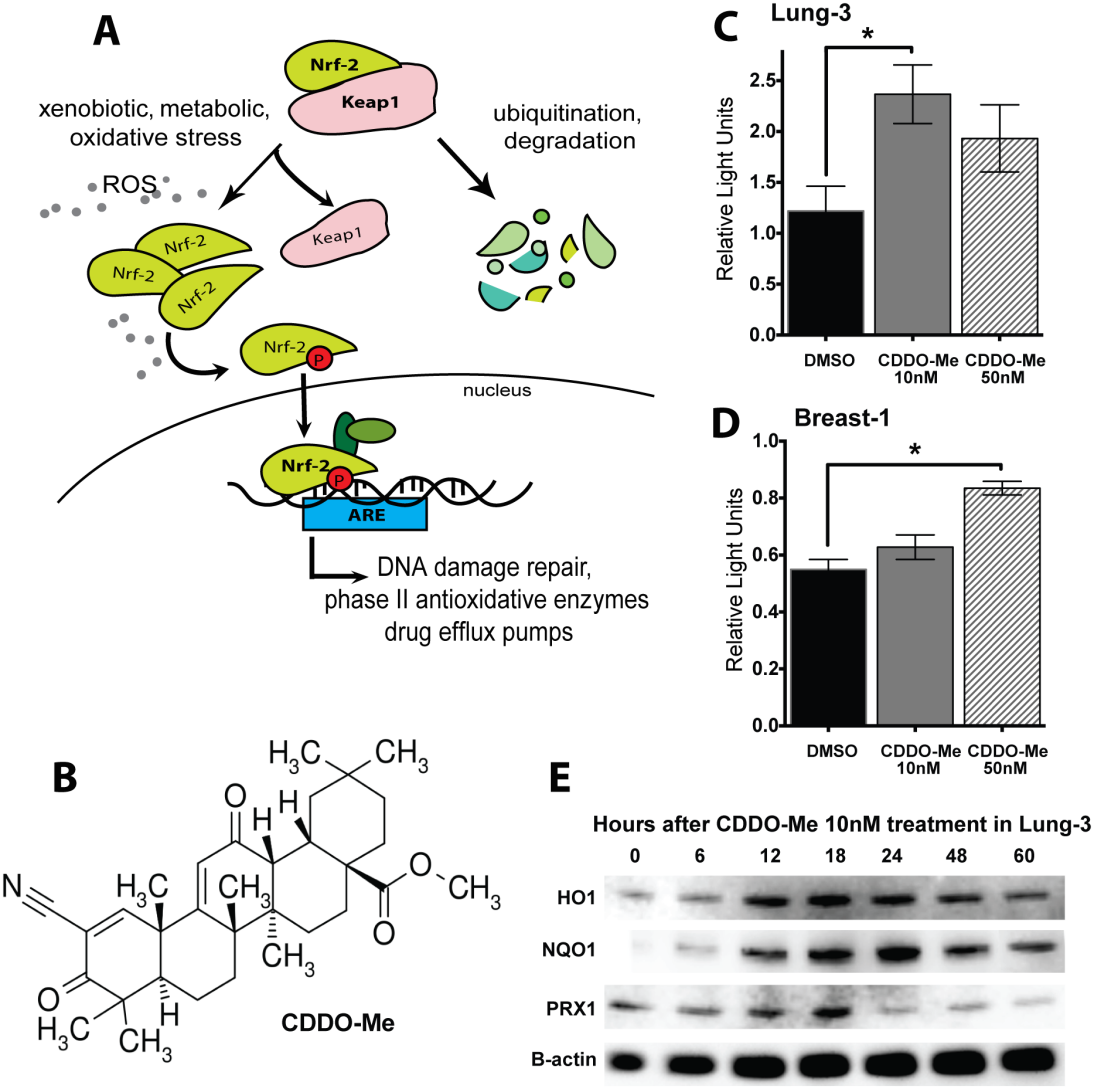
CDDO-Me activates the Nrf2 antioxidant pathway in epithelial cells. (A) Nrf2 Pathway: Nrf2 is a transcription factor normally bound by its cytoplasmic repressor Keap1, which acts as a molecular oxidative sensor and marks Nrf2 for degradation. When there is an abundance of reactive species in the cells, Nrf2 accumulates in the cytoplasm, eventually undergoing various phosphorylation events to translocate to the nucleus and bind to Antioxidant Response Elements (AREs) in the genome, resulting in the transcription of multiple antioxidative and cyto-protective genes. CDDO-Me acts by facilitating the dissociation between Keap1 and Nrf2, leading to Nrf2 activation. (B) Chemical structure of CDDO-Me: Oleana-1,9(11)-dien-28-oicacid, 2-cyano-3,12-dioxo-, methyl ester (RTA-402; bardoxolone-methyl). (C, D) CDDO-Me increases expression of ARE-driven luciferase 18 hours after drug treatment in HBEC 3KT and HME1, respectively. Firefly ARE-luciferase normalized to renilla control (RLU). Mean ± SEM of 6 replicates, *p<0.05 using paired t-test (between DMSO and drug). (E) CDDO-Me 10 nM activates heme oxygenase-1 (HO1, band observed at ∼32 kDa), NADPH dehydrogenase quinone (NQO1, band observed at ∼30 kDa), and peroxiredoxin (PRX1, band observed at ∼20 kDa), all downstream targets of Nrf2/ARE and peaking at approximately18 hours after treatment in HBEC 3KT.

A central cellular mechanism for dealing with oxidative stress, including response to radiation, is through induction of the Nrf2/Antioxidant Response Element (ARE) pathway, which is responsible for detoxifying cellular insults. Nrf2 is a transcription factor that is normally bound by its cytoplasmic repressor Keap1, which acts as a molecular oxidative sensor. When the level of reactive species in a cell reaches a certain threshold, it changes cysteine residues on Keap1, inhibiting the ubiquitination and subsequent degradation of Nrf2. Newly synthesized Nrf2 is then unable to interact with Keap1, resulting in Nrf2 accumulation and phosphorylation until it translocates to the nucleus, where it binds to AREs in the genome. This results in transcription of multiple antioxidative and cytoprotective genes (Fig. 1A) [6]. Interestingly, the Nrf2 pathway is commonly dysregulated in cancers, providing tumors added detoxifying potential against cellular insults [7]–[9]. To level the playing field and protect normal tissues post-IR, new therapeutic agents that enhance repair and neutralize ROS to mitigate the negative effects of radiation are needed. However, in order for these agents to be realistically efficacious, they cannot provide the same level of protection to cancerous cells.

The synthetic triterpenoid CDDO-Me (oleana-1,9 (11)-dien-28-oicacid, 2-cyano-3,12-dioxo-, methyl ester; bardoxolone-methyl) is a multifunctional and largely nontoxic antioxidant, anti-inflammatory modulator with the ability to activate cytoprotective pathways (Fig. 1B). This orally available drug can increase the activity of Nrf2/ARE in the low nanomolar range (S1 Fig.) [10], [11]. As the concentration of CDDO-Me increases into the micromolar range, it can induce differentiation and inhibit cell proliferation, eventually leading to cell death via apoptosis through IKK and NF-κB pathways [12]. CDDO-Me has shown antitumor activity in lymphoma patients in a phase I human trial and prevents formation of estrogen receptor-negative mammary tumors in mouse models of breast cancer [13], [14]. Additionally, the ethylamide analogue of CDDO (CDDO-Ea) can prevent cancer progression in mouse models of lung and prostate cancer [15], [16]. Additional work by the Liby and Sporn group show that CDDO compounds activate Nrf2 downstream effectors, such as heme oxygenase-1 (HO1), as well as other pathways in both transgenic and wildtype mouse models [14], [17], [18].

The radioprotective effects of triterpenoids have been well established. At doses that inhibit NF-κB, a CDDO analog significantly increases survival and decreases apoptosis in different tissues of lethally irradiated zebrafish embryos [19]. CDDO-Ea has been demonstrated to stabilize and activate Nrf2 in the colons of mice exposed to acute lethal doses of ionizing radiation resulting in increased survival [20]. Furthermore, CDDO-Me has been shown to be an effective radioprotector in human colonic epithelial cells [20], [21].

The purpose of the current study was to determine the radioprotective effects of CDDO-Me in normal, non-cancerous human bronchial (HBECs) and mammary (HMECs) epithelial cells as well as in human lung and breast cancer cell lines. We established that while low dose CDDO-Me protected both normal HBECs and HMECs through an Nrf2-dependent mechanism, CDDO-Me provided no further induction of Nrf2 in human lung and breast cancer cells, nor did it protect these cancer cells against radiation-induced apoptosis. These results demonstrate that this orally available non-toxic radioprotector may have important medical implications for patients undergoing radiotherapy as well as individuals who might be exposed to occupational radiation.

## Methods and Materials

### Cell Culture

#### Human lung - bronchial epithelial cells

Human bronchial epithelial cells (HBECs) were obtained from central lung bronchi and immortalized using ectopic expression of human telomerase reverse transcriptase (hTERT; T) and cyclin-dependent kinase 4 (CDK4; K) as described previously [22]. Experimentally transformed HBECs used in the present studies included overexpressing *KRas* ^V12^, *p53* knockdown via shRNA, and *myc* overexpression as previously described [23]. Immortalized non-cancerous HBEC 3KT, HBEC 30KT, and the experimentally transformed HBECs were cultured at 37°C in 5% CO_2_ in Keratinocyte Serum Free Media (KSFM) (Gibco) containing 50 µg/mL of bovine pituitary extract and 5 µg/mL of epidermal growth factor on porcine gelatin-coated tissue culture dishes (Sigma Aldrich).

Human breast - mammary epithelial cells: Human mammary epithelial cells (HME1) were immortalized by retroviral infection with hTERT and have a normal diploid karyotype (ATCC Cell Systems, Gaithsburg, MD). HMEC50 cells were originally derived from the noncancerous breast tissue of a female diagnosed with Li-Fraumeni syndrome (TP53 heterozygous) as previously described [24]. All HMECs were cultured in serum-free conditions as previously described and were mycoplasma free and DNA fingerprinted [25].

#### Human cancer cell lines

Non-small cell lung cancer (NSCLC) cells A549, H2009, HCC 2429, HCC 4017, H23, and HCC 15 were supplied by John Minna (Hamon Cancer Center, UT Southwestern Medical Center, Dallas, TX). The breast cancer cell line MDA-MB-231 was kindly provided by Michael White (Department of Cell Biology, University of Texas Southwestern Medical School, Dallas, TX). All cancer cell lines were cultured in basal medium supplemented with 10% Cosmic Calf Serum (Thermo Scientific) at 37°C in 5% CO_2_. All cell lines used in the present studies were mycoplasma free (e-Myco kit, Boca Scientific) and DNA fingerprinted (PowerPlex 1.2, Promega). All cells were compared to the complete database in our own collection and to that of ATCC. All cell lines are commercially available through the ATCC Cell Systems (Gaithersburg, MD).

#### Human lymphocytes

Peripheral blood mononuclear cells (PBMCs) were isolated via centrifugation from the buffy coat of whole blood donated by healthy human volunteers via venipuncture. Informed consent was obtained from each donor in accordance with the Declaration of Helsinki and approved by the Institutional Review Board at UT Southwestern Medical Center (Dallas, TX). Cells were stimulated using 1 ug/mL Lectin, PHA-L (EMD Biosciences) and cultured in suspension of RPMI-1640 media (Gibco) supplemented with 10% Cosmic Calf Serum (Thermo Scientific) at 37°C in 5% CO_2_.

#### Mouse embryonic fibroblast (MEF) cells

*Nrf2-*heterozygous (+/−) and *nrf2*-deficient (−/−) cells were the generous gift of Ralph DeBerardinis (Children’s Medical Center Research Institute, UT Southwestern Medical Center, Dallas, TX) [26]. Cells were cultured in basal medium supplemented with 10% Cosmic Calf Serum (Thermo Scientific) at 37°C in 5% CO_2_.

### Genetic Manipulations

#### Nrf2 knockdown

Stable Nrf2 knockdown cells lines were established by infecting epithelial cells (HBEC 3KT and HME1) with a validated anti-Nrf2 shRNA expressing lentiviral vector (pGIPZ, OpenBiosystems) in the presence of 2 µg/mL Polybrene (Sigma).

### Drug Treatment and Radiation

CDDO-Me (Reata Pharmaceuticals, Irving, TX) was dissolved in DMSO. Subconfluent cell cultures were treated with 10, 50, or 150 nM CDDO-Me. Experimental concentrations of CDDO-Me were determined based on cell toxicity for the different cell types (shown in S2 Fig.) and used at the lowest effective dose for each tissue type-cell. Drug was administered 18 hours prior to gamma radiation exposure using a ^137^Cs source at 243.08 cGy/min (University of Texas Southwestern Medical Center with dosimetry provided by physicists in the Department of Radiation Oncology). Control experiments were treated with solvent only.

### ARE-Luciferase Reporter

Cells were co-transfected with pGL4.37 [*luc2*/ARE/hygro], and pGL4.73 [*hRluc*/SV40] as a transfection expression control using 3∶1 FuGENE HD according to the manufacturer’s instructions (Promega). Briefly, cells were treated with drug 18 hours after luciferase transfection, and luciferase activity was measured using Dual-Glo Luciferase Assay (Promega) after another 18 hours. Each ARE-firefly luciferase value was normalized against Renilla luciferase.

### Colony Formation Assays

Immediately following IR exposure, cells were trypsinized and seeded in triplicate in 10-cm dishes at clonogenic density (ranging from 100–1000 cells per dish) for colony formation assays. Ten days later, dishes were stained with a mixture of 6.0% glutaraldehyde and 0.5% crystal violet, and colonies (defined as clusters of >50 cells) were counted. Cell survival measurements were fitted using a linear quadratic equation [*SF* = exp (−α*D* − β*D* ^2^)] (SF: surviving fraction; D: radiation dose in Gy) using GraphPad Prism, and dose-modifying factors (DMF) calculated for each as a measure of radioprotection as described [27]. DMF less than 1.2 was considered the cutoff for significant protection.

### Comet Assay

Alkaline comet assay (Trevigen) to detect DNA damage at 30 minutes post-IR was performed according to manufacturer’s instructions. Twenty fields (200x magnification) were scanned continuously under a fluorescence microscope. Approximately 50 cells per condition were analyzed using OpenComet software [28]. Tail moment [tail length × tail DNA %] and tail DNA % values generated by OpenComet were analyzed as a measure of DNA damage.

### Western Blot Analysis

Cells were lysed in Laemelli SDS reducing buffer [50 mM Tris-HCl (pH 6.8), 2% SDS, and 10% glycerol], boiled, and separated by SDS/PAGE. The following antibodies were used: anti- HO1, -PRX1, -NQO1 (1∶1000; AbCam), anti- Nrf2 (1∶500 Santa Cruz; 1∶1000 Cell Signaling), anti-phospho-Nrf2 (1∶5000; AbCam), and anti–β-actin (1∶20,000; Sigma).

### Proliferation Assay

MEFs were treated with 50 nM CDDO-Me 18 hours prior to 10 Gy gamma radiation and counted at various times thereafter using an automated cell counter (TC20, Biorad) in the presence of typan blue to assess cell viability.

### Viability Assays

CDDO-Me or DMSO was added to cells at 60% confluency, and cell viability was determined 48–60 hours later with CellTiter-Glo (Promega) as per manufacturer’s protocols. Reported median lethal concentration (LC_50_) values are based on the average of two experiments with 6 replicates and calculated from dose-response curves generated with nonlinear regression in GraphPad Prism 6 (GraphPad Software, Inc).

### Statistical Methods

All significance values are p<0.05, unless otherwise stated, and were calculated using two-sided t-tests between the treatment group and its appropriate control.

## Results

### CDDO-Me induces the Nrf2 pathway in non-cancerous HBECs and HMECs, but not breast and lung cancer cell lines

To confirm that CDDO-Me (Fig. 1B) activates the Nrf2 pathway in the cells used, HBEC 3KT (Lung-3) and HME1 (Breast-1) transfected with the ARE-luciferase reporter were treated with CDDO-Me or DMSO. After 18 hours, CDDO-Me 10 nM significantly increased luciferase expression in lung, and 50 nM increased luciferase expression in breast (p<0.05, paired t-test compared to DMSO control) (Fig. 1C, D). NSCLC cells tested, however, did not have increased ARE-luciferase after treatment with CDDO-Me (S3 Fig.). Additionally, protein lysates collected at various times after CDDO-Me 10 nM treatment of normal Lung-3 cells showed an increase of Nrf2/ARE downstream targets, including heme oxygenase (HO1), NADPH dehydrogenase quinone (NQO1), and peroxiredoxin (PRX1) (Fig. 1E; S1 Fig.). Expression of these downstream enzymes peaks around 18 hours. For this reason, an 18-hour pre-treatment with CDDO-Me was used for all subsequent radioprotection experiments.

### Pre-treatment with CDDO-Me decreases IR-induced DNA damage in bronchial and mammary epithelial cells as well as in PBMCs

**Figure 2:**
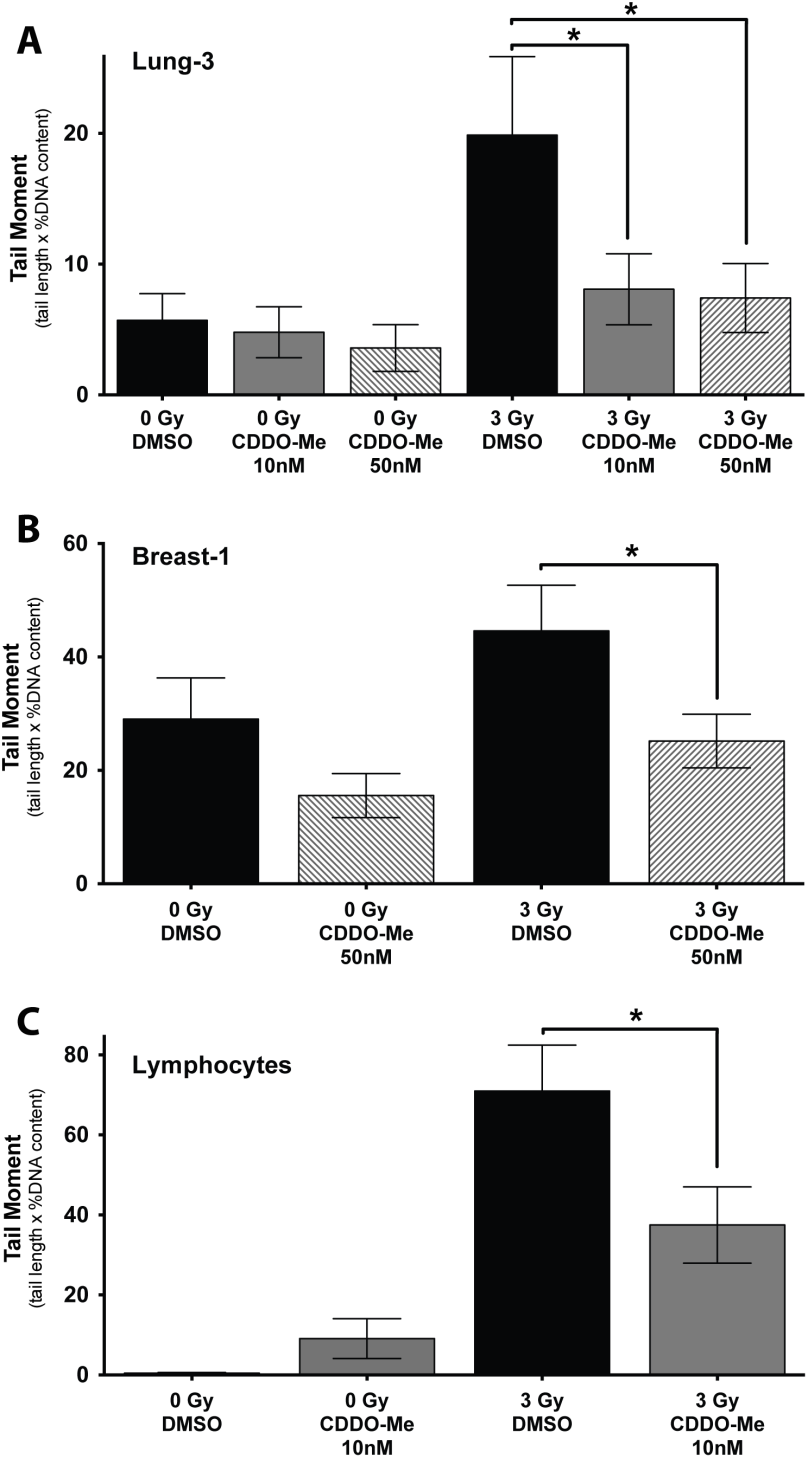
Pre-treatment with CDDO-Me decreases IR-induced DNA damage in a variety of non-cancerous cells. CDDO-Me decreases radiation-induced DNA damage in the alkaline comet assay in bronchial and mammary epithelial cells as well as human lymphocytes. (A) HBEC 3KT, (B) HME1, and (C) PBMCs were treated with CDDO-Me 18 hours prior to IR, then mounted on slides 30 min post-IR. Data analyzed and calculated using Open Comet software [tail moment = tail length x tail DNA percentage]. Mean ± SEM of >50 cells per condition, *p<0.05 using t-test (compared to 3 Gy DMSO). **p<0.01, using T-test (compared to 0 Gy DMSO).

Alkaline comet assays were performed on lung and breast epithelial cells 30 minutes after radiation to determine if CDDO-Me protected against IR-induced DNA damage. Since many of the adverse effects of radiation occur in the blood, peripheral blood mononuclear cells (PBMCs) were assessed to determine if CDDO-Me also rescued human lymphocytes against IR-induced DNA damage. We found that pre-treatment with CDDO-Me protected all three non-cancerous cell types against radiation-induced DNA damage as seen by significantly decreased tail moments using the alkaline comet assay in PBMCs (Fig. 2C) as well as HBEC 3KT and HME1 (Fig. 2A, B) (*p<0.05, t-test compared to 3 Gy DMSO control). The partial protection of human lymphocytes with CDDO-Me is particularly important since significant hematological toxicities are associated with radiation therapy for lung and breast cancers [29].

### CDDO-Me is a significant radioprotective countermeasure in normal epithelia

**Figure 3:**
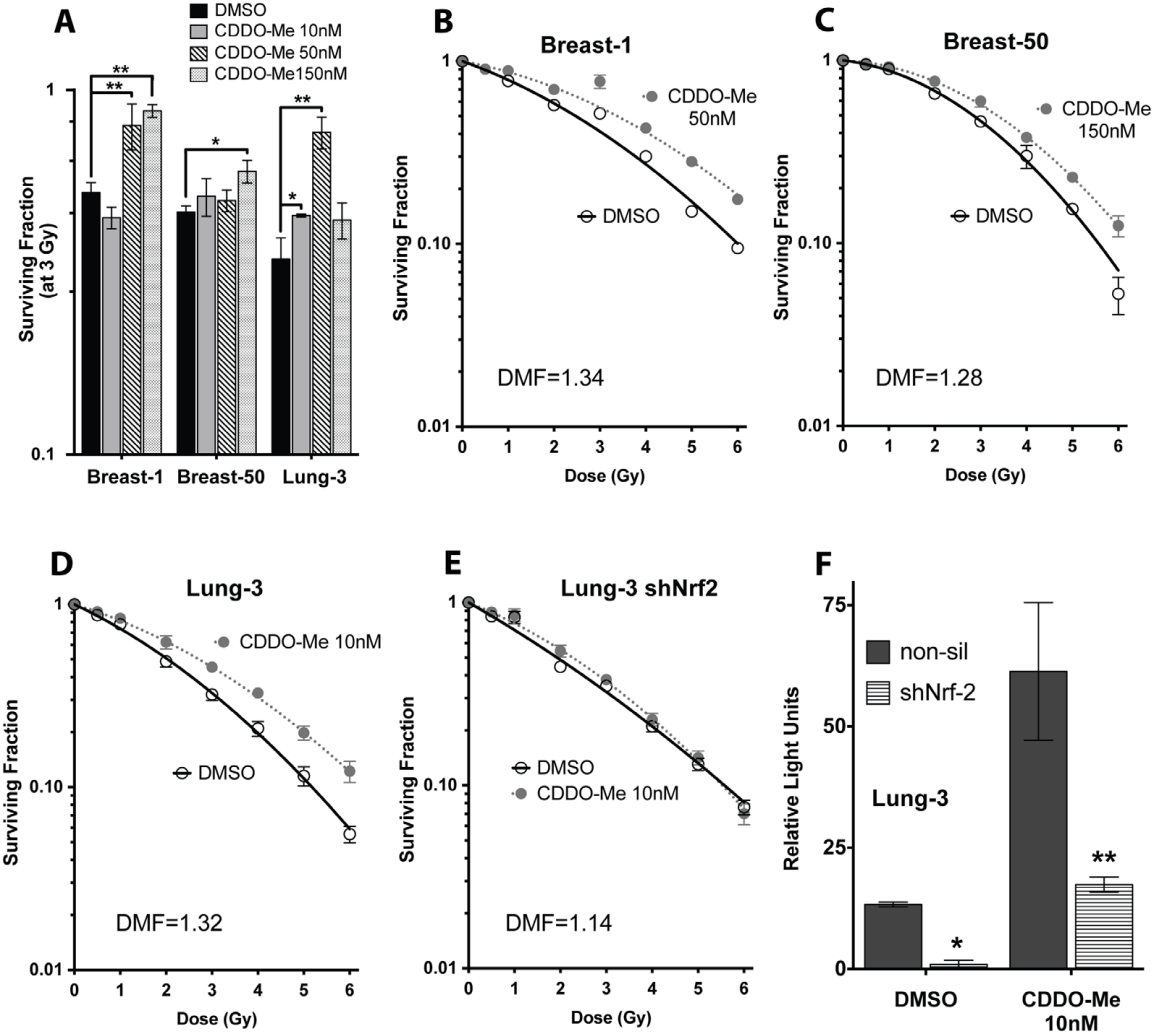
CDDO-Me is a potent radiation countermeasure in bronchial and breast epithelial cells, and Nrf2 knockdown abrogates these radioprotective effects. (A) Normal breast and lung epithelia are radioprotected at multiple doses of CDDO-Me. Cells were treated with drug 18 hours before exposure to 3 Gy gamma IR, then seeded immediately into clonogenicity. Colonies grown for ∼14 days before fixation with 6% glutaraldehyde/0.5% crystal violet stain. Mean ± SEM of four experiments seeded in triplicate, *p<0.05, **p<0.001 using t-test (compared to DMSO at 3 Gy). (B, C) HMEC and (D) HBEC cells pre-treated with 10 nM CDDO-Me have a significant increase in clonogenic survival. (E) HBEC 3KT with sh-Nrf2 see no radioprotection when pre-treated with CDDO-Me. Clonogenic survivals, mean ± SEM with linear-quadratic fit curve of four experiments seeded in triplicate. (F) Nrf2 knockdown cells have a ∼90% decrease of Nrf2 activity compared to non-silencing control, with diminished basal and CDDO-Me-induced ARE-luciferase activity. Mean ± SEM of six replicates, *p<0.05, **p<0.01, t-test (compared to non-silencing control).

**Figure 4:**
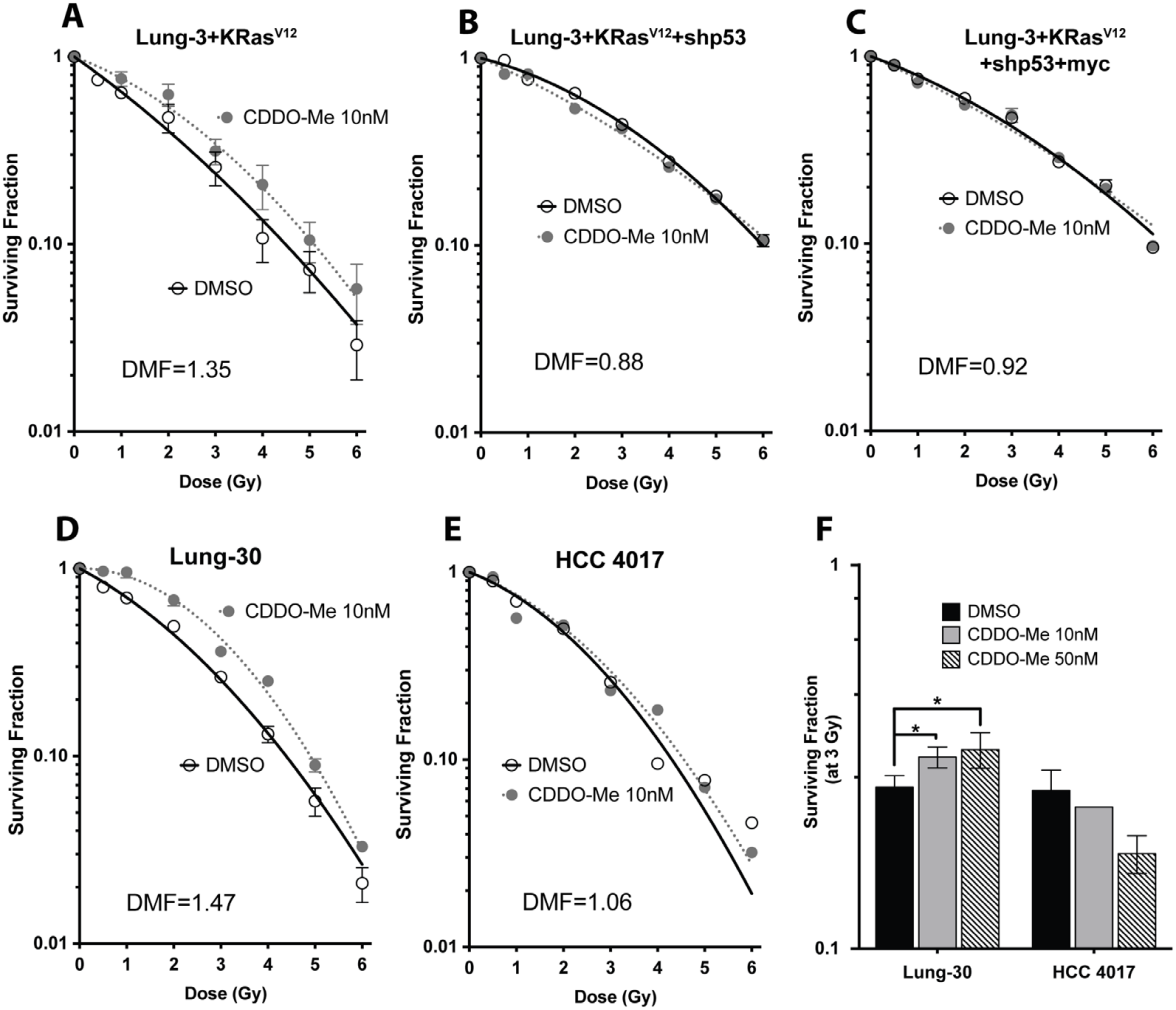
CDDO-Me radioprotection decreases with progressive oncogenic manipulations in HBECs and in a matched NSCLC line. Isogenic oncogenic progression in HBEC 3KT. Immortalized HBECs with (A) lenti-*KRas* ^V12^, (B) lenti-*KRas* ^V12^ and sh*p53* knockdown, and (C) lenti-*KRas* ^V12^, sh*p53*, and *myc* overexpression. Only lenti-*KRas* ^V12^ cells are still moderately protected by CDDO-Me, but further oncogenic changes eliminate the radioprotective effects of CDDO-Me. (D) HBEC 30KT are protected by CDDO-Me. (E) HCC 4017, a NSCLC isolated from the same patient from which HBEC 30KT was derived, are unprotected by CDDO-Me. (F) Increasing concentrations to 50 nM still enhances clonogenic survival of HBEC 30KT, but actually seems to decrease survival in HCC 4017 after 3 Gy radiation. Mean ± SEM of three experiments seeded in triplicate, **p<0.01, t-test (compared to DMSO at 3 Gy).

To determine the potential radioprotective effects of CDDO-Me, clonogenic survival assays post-IR was assessed in multiple immortalized but non-cancerous bronchial (HBEC 3KT, 30KT) and breast (HME1, HMEC 50) epithelial cells. Since epithelial cells are more sensitive to the cytotoxic effects of CDDO-Me compared to other malignant cell types (S2 Fig.), normal breast and lung cells were pre-treated with low nanomolar concentrations before exposure to 3 Gy radiation to determine the lowest effective radioprotective dose (10–50 nM for lung, 50–150 nM for breast) (Fig. 3A; HBEC 30KT in Fig. 4F). Both cell types, when exposed to CDDO-Me 18 hours prior to IR, had an increase in clonogenic survival when compared to DMSO treated cells (HBEC 3KT, DMF = 1.32; HBEC 30KT, DMF = 1.47; HME1, DMF = 1.34; HMEC 50, DMF = 1.28) (Figs. 3B–D and Fig. 4D). The DMFs observed with CDDO-Me are greater than most standard radioprotective agents currently used, including amifostine [30], [31]. This demonstrates that CDDO-Me is a potent radioprotective agent when given before IR in lung and breast epithelial cells.

### Nrf2 knockdown eliminates radioprotective effects of CDDO-Me

To confirm that Nrf2 is the mechanism through which CDDO-Me protects epithelial cells, clonogenic survival post-IR was assessed in cells stably expressing Nrf2 shRNA (shNrf2). Lung-3 cells with shNrf2 knockdown are not significantly radioprotected by CDDO-Me pretreatment (DMF = 1.14) (Fig. 3E), whereas cells with intact Nrf2 have increased survival when treated with CDDO-Me (Fig. 3D; data for breast not shown). Nrf2 knockdown cells have decreased basal and induced expression of Nrf2 as evidenced by ARE-luciferase reporter expression when compared to an shRNA non-silencing control (*p<0.05, **p<0.01, t-test) (Fig. 3F). This indicates the Nrf2 pathway is integral to CDDO-Me radioprotection in normal epithelia.

As additional evidence that Nrf2 is necessary for CDDO-Me radioprotection, survival and viability after a sub-lethal doses of IR was assessed in *nrf2*-deficient or *nrf*-heterozygous mouse embryonic fibroblasts. Pretreatment with CDDO-Me increased the percentage of viable *nrf2*+/− cells 48 hours post-IR, but did not protect *nrf2*−/− cells (S4-A Fig.). Additionally, cells with deficient *nrf2* die faster compared to heterozygous cells (S4-B Fig.). These findings further corroborate the notion that Nrf2 is necessary for both responses to radiation as well as protection by CDDO-Me.

### Oncogenically progressed HBECs, NSCLCs, and breast cancer cells are not protected by CDDO-Me

In order to determine if experimentally cancer progressed human epithelial cells and cancer cell lines are also protected by CDDO-Me, clonogenic survival post-IR was assessed using an isogenic series of cell lines with progressive oncogenic manipulations. HBEC 3KT with *KRas* overexpression were still protected from radiation with CDDO-Me (Lung-3+lenti-*KRas* ^V12^, DMF = 1.35) (Fig. 4A). When additional changes were introduced, including *p53* knockdown and *myc* overexpression, protection from CDDO-Me was lost (Lung-3+lenti-*KRas* ^V12^+sh*p53*, DMF = 0.88; Lung-3+lenti-*KRas* ^V12^+sh*p53*+*myc*, DMF = 0.92) (Fig. 4B, C).

To further show that CDDO-Me only protects non-malignant cells, we performed clonogenic survivals in a lung cancer line (HCC 4017), which has a matched HBEC (Lung-30) derived of normal, non-cancerous tissue from the same patient. Importantly, while normal Lung-30 was protected by 10 nM CDDO-Me (HBEC 30KT, DMF = 1.47) (Fig. 4D), the tumor cell line from the same patient was not protected (HCC 4017, DMF = 1.06) (Fig. 4E). Furthermore, increasing the concentration to 50 nM CDDO-Me decreases survival after radiation to HCC 4017 cells while still providing radioprotection to Lung-30 cells (Fig. 4F). This is a promising result since CDDO-Me appears to specifically provide protection to normal, noncancerous human cells, thus supporting the use of such radioprotectors prior to radiation therapy for cancer patients.

**Figure 5:**
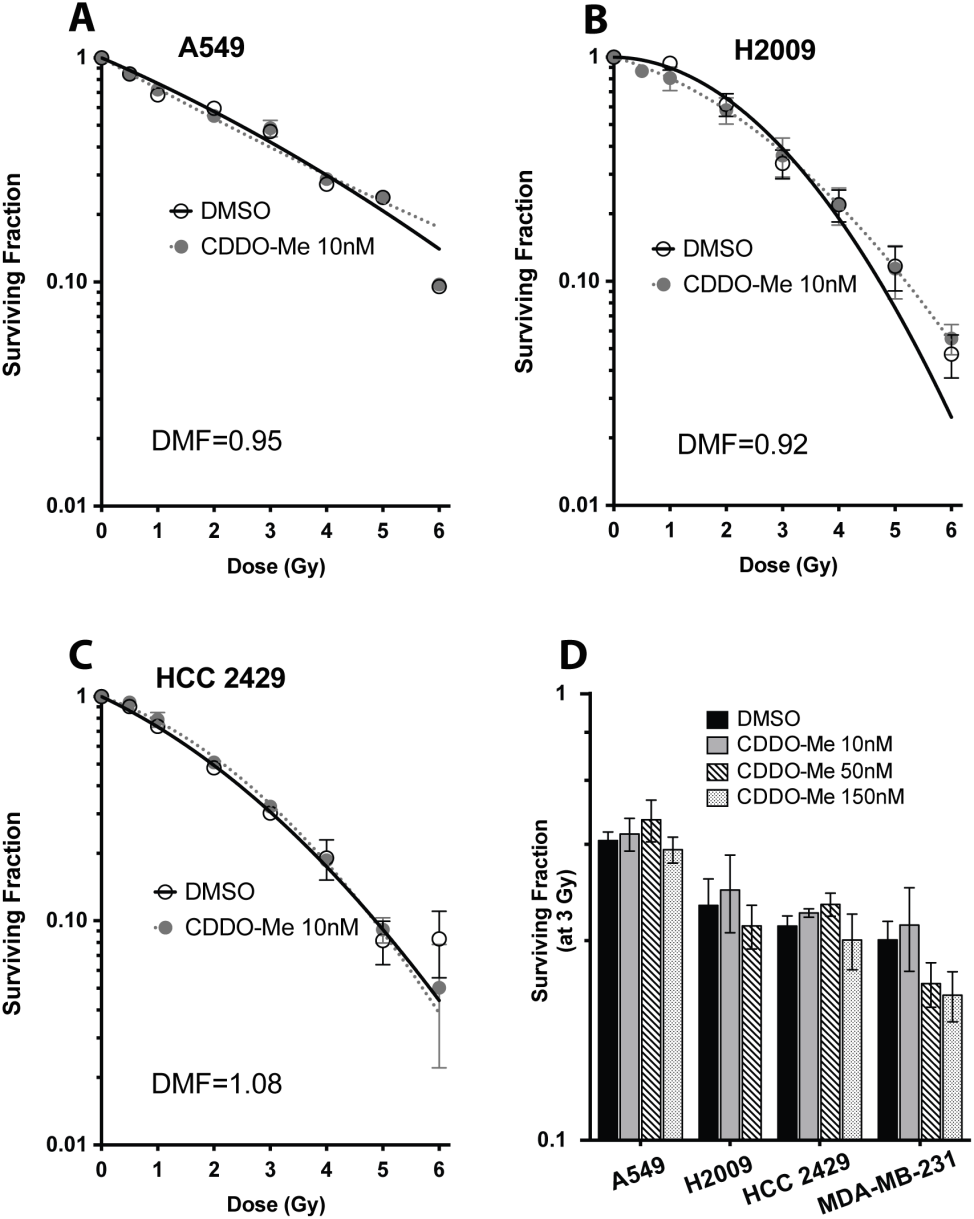
NSCLC and breast cancer cells are not protected with CDDO-Me. Clonogenic survivals show that (A) A549, (B) H2009, and (C) HCC 2429 are not protected when pretreated with the same concentration of CDDO-Me (10 nM) that protected HBEC cells. (E) Even higher concentrations of CDDO-Me are not protective of cancer cells after 3 Gy radiation, including MDA-MB-231 breast cancer line. However, 150 nM CDDO-Me significantly decreases the clonogenic survival of MDA-MD-231 cells after exposure to 3 Gy radiation. Mean ± SEM of three experiments seeded in triplicate, **p<0.01, t-test (compared to DMSO at 3 Gy).

We also tested various other NSCLC cells and a breast cancer cell line for potential radioprotection with CDDO-Me. The basal radiosensitivity (SF_2_) increases in Lung-3 (HBEC 3KT) with each additional oncogenic manipulation, indicating that these cells become more radio-resistant during the stepwise mutations that lead to cancer, whereas Lung-30′s (HBEC 30KT) matched tumor line is actually more sensitive to radiation (Table 1). Since NSCLCs are heterogeneous in their radio-responsivity, we tested a range of radio-sensitive and resistant lines (indicated by the surviving fraction of cells at 2 Gy [SF_2_]), as well as NSCLCs containing a variety of different mutations (Table 1). NSCLCs pretreated with the same concentration of CDDO-Me that protected normal lung epithelial cells (10 nM) were not protected from radiation, regardless of radiosensitivity or mutation status (A549, DMF = 0.95; H2009, DMF = 0.92; HCC2429, DMF = 1.08; data for HCC 15 and H23 not shown) (Fig. 5A–C). This indicates that multiple oncogenic alterations have an effect of both radiation response as well as protection by CDDO-Me.

**Table 1:** Panel of cell radiosensitivity and mutation status.

Cell Line	SF_2_	*KRas*	*p53*	*Keap1*
**HBEC 3KT**	**0.487**	wt	wt	wt
**HBEC 3KT +** ***KRas^ V12^***	**0.474**	**X**	wt	wt
**HBEC 3KT** **+** ***KRas^ V12^*** **+sh** ***p53***	**0.650**	**X**	**X**	wt
**HBEC 3KT** **+** ***KRas^V12^*** **+sh** ***p53*** **+** ***myc***	**0.702**	**X**	**X**	wt
**HBEC 30KT**	**0.442**	wt	wt	-
**HCC 4017**	**0.321**	**mut**	**mut** (C833A)	wt
**A549**	**0.771**	**mut** (G34A)	wt	**mut** (G333C);constitutive Nrf2 activation
**H2009**	**0.615**	**mut** (G35C)	**mut** (G818T)	wt
**HCC 2429**	**0.514**	wt	**mut** (G800A)	wt
**HCC 15**	**0.484**	wt	**mut** (A776T)	wt
**H23**	**0.053**	**mut**	**mut**	**mut**; Nrf2 still inducible
**HME1**	**0.528**	wt	wt	wt
**HMEC 50**	**0.659**	wt	**mut**	-
**MDA-MB-231**	**0.452**	**mut** (G38A)	**mut** (G839A)	wt

A summary of all cell lines used in the present study. Surviving fraction of cells at 2 Gy (SF_2_) is used as a metric of radio-sensitivity, with SF_2_>0.6 considered a “resistant” line and SF_2_<0.4 considered a “sensitive” line. Mutation status of *KRas*, *p53*, and *Keap1/Nrf2* is listed as either wildtype (wt) or mutated (mut) as determined by full exon sequencing (John Minna and Adi Gazdar, UT-Southwestern Medical Center, Dallas, TX, personal communications). A mutation is present in Keap1 in the NSCLC H23 cell line (personal communications with Brandon Probst, Reata Pharmaceuticals). “X” indicates experimentally manipulated gene expression.

Since cancer cell lines can generally survive in higher concentrations of CDDO-Me when compared to normal epithelial cells (S2 Fig.), we also treated the malignant cells with higher concentrations of CDDO-Me to confirm that cancer cells would not be protected at higher doses of CDDO-Me. Even concentrations up to 150 nM were not sufficient to protect NSCLC, including HCC 15 and H23 (data not shown), nor did it protect MDA-MB-231, a breast cancer cell line (Fig. 5D). This demonstrates that the same low nanomolar concentrations of CDDO-Me that protect normal epithelial cells are highly unlikely to be protective in malignant cells.

## Discussion

When cancer patients undergo radiation therapy, the relationship between radiation dose and tumor response generally follows a dose-response curve. Unfortunately, normal tissue damage follows an even steeper increase with increasing radiation dose [32]. Long-term effects and toxicity for the patient caused from normal tissue damage limit the total dose that can be administered, and for this reason, widening the therapeutic margin has been and remains a crucial goal in the radiation oncology field. In this study, we show that CDDO-Me selectively protects normal non-cancerous lung and breast epithelial cells while leaving tumor cells unprotected against radiation, resulting in a potentially higher therapeutic window for current standards of care radiotherapy.

In order for a radioprotector to be classified as such, or to be used with conventional radiotherapeutic doses, it is critical that the agent be able to be administered in optimal dosing, have low toxicity, and most importantly, not protect tumor cells. The current standard for acute radiation exposure is amifostine, a hydrophilic phosphorothioate compound that does not readily cross cell membranes, must be converted to an active metabolite, and can only be administered intravenously [30]. The radioprotection amifostine provides varies greatly depending on the oxygen content and tissue type, with lung protection factors being amongst the lowest (DMF = ∼1.2). In addition, amifostine has high cytotoxic activity against normal cells and has serious side effects such as hypotension and neuropathies [30]. In contrast, we found that CDDO-Me is much more effective in protecting both normal lung and breast epithelial cells (average DMF = ∼1.35). Since CDDO-Me is orally available with a low toxicity profile, this makes it a more attractive option as a radioprotector, especially when only given short term.

Not only is CDDO-Me a potent radioprotective countermeasure in epithelial cells, but we show in this study that CDDO-Me can significantly protect human lymphocytes from radiation-induced DNA damage. This is a particularly promising result considering that damage to the hematopoietic system is often one of the main dose-limiting toxicities of radiation therapy, with anemia, bleeding, and infections being common [29]. Furthermore, the long-term negative consequences of radiation include development of secondary leukemia and lymphomas later in life [33]. Since we demonstrate that CDDO-Me has radioprotective effects against human blood lymphocytes, this is one more added benefit of CDDO-Me that may help protect persons exposed to radiation.

Since Nrf2 is necessary for CDDO-Me to exert its protective effects on epithelial cells, it is necessary to point out that even cells with Nrf2 knockdown have a small amount of Nrf2 activity, and these cells are still induced by CDDO-Me. Similar effects have been observed in other studies [20], but since there is never a 100% decrease of Nrf2 with shRNA knockdowns, there may be residual Nrf2 even in the sh-Nrf2 cells. Since the Nrf2 protein is extremely difficult to assay directly, the exact quantification of knockdown level is determined either through quantitative RT-PCR or Western Blot of downstream markers (∼60% knockdown, data not shown), or using a reporter, such as the ARE-luciferase (∼90% knockdown, shown in Fig. 3F). Since there is still some Nrf2 leftover in these cells, this may partially explain why the Nrf2/ARE pathway is still partially inducible by CDDO-Me in knockdown cells, but this induction may not be sufficient to exert a protective effect. To confirm the importance of the Nrf2 signaling pathway in the radioprotection observed, we demonstrate that mouse cells with complete *nrf2*-deficiency are unprotected by CDDO-Me. It is important to point out that CDDO-Me is likely activating other additional compensatory pathways.

When radiation exposure produces large amounts of reactive species in cells, Nrf2/ARE is not the only pathway activated. Radiation has been shown to stabilize hypoxia inducible factor (HIF-1α) by activating p38 MAPK and resulting in the decreased half-life of its E3 specific ligase, protein von Hippel-Lindau [34]. There have been reports that amifostine induces HIF-1α in both cell culture and mouse tissues [35]. Thus, reactive species produced by radiation may mimic and affect multiple pathways simultaneously, including the Nrf2/ARE and HIF/HRE pathways.

Although CDDO-Me is a potent radioprotector for normal, non-cancerous cells, it did not protect any of the cancer cells tested in these studies. Interestingly, c-myc has been identified as an Nrf2-interacting protein [36], but a single mutation is unlikely responsible for loss of CDDO-Me effects. This is clearly demonstrated with the experimentally manipulated gene expression in the isogenic HBEC system–immortalized HBECs with lenti-*KRas* ^V12^ and sh*p53* knockdown are not protected regardless of whether or not the cells have *myc* overexpression. Additionally, some of the NSCLC cells with intact *KRas* or *p53* yet are not protected by CDDO-Me, indicating that multiple oncogenic changes are required to confer resistance to CDDO-Me radioprotection.

There are published reports showing that higher doses of CDDO-Me and other triterpenoids (above 1 µM) can inhibit cancer cell growth and induce cancer cell death in a multitude of cancer types [16], [37]. The flip side, however, is that these higher doses also inhibit the growth and affect the viability of normal cells (S2 Fig.). In the nanomolar range used in these experiments (up to 150 nM), we did not observe any decreases in proliferation or increased cell death in NSCLC cell lines in the absence of radiation treatment that would be expected at higher concentrations. While we do not show any significant chemo-preventative effects of CDDO-Me in the lung, there are indications slightly higher doses (>150 nM) of CDDO-Me may act as a radiosensitizer in some lung and breast cancer cells. Most promisingly, we did not observe any radioprotective effects in cancer cells, even when the doses were increased.

The original phase II clinical trial using CDDO-Me for treatment of diabetic kidney disease used doses ranging from 25–150 mg daily [38]. While these doses are not toxic as a one-time treatment, they have the potential to accumulate over time as almost all patients experienced some side effects over the course of 52 weeks [38]. However, our present series of experiments utilized low nanomolar concentrations of CDDO-Me as a one-time treatment, allowing patients to conceivably be treated for a short period before radiation exposure and minimizing potential long-term toxicities.

CDDO-Me, and other compounds in the same triterpenoid family, have been shown to have chemoprotective properties in addition to radioprotective properties [39]. Many chemotherapeutic drugs used for lung cancer, such as paclitaxel and carboplatin, induce DNA damage and produce ROS; these effects can be detrimental to healthy non-cancerous cells. Damage to rapidly dividing cells (bone marrow, gastrointestinal tract, and skin) often results in radiation-induced toxicities. For this reason, the use of CDDO-Me could be expanded as a potentially effective chemoprotective agent. Ideally, CDDO-Me can be given short-term to cancer patients undergoing radiation or chemotherapy to increase the therapeutic margin, resulting in better outcomes and less toxicity.

## Supporting Information

S1 Fig **CDDO-Me increases Nrf2 protein over time. (A)** Protein levels of phosphor-Nrf2 (band observed at ∼120 kDa) and total Nrf2 (bands observed at ∼68, 75 kDa) after treatment with 10 nM CDDO-Me in HBEC 3KT.
(TIFF)

S2 Fig **Epithelial cells are more sensitive to CDDO-Me when compared to cancer cells.** Cell Titer Glo toxicity curves of various (A) NSCLCs and (B) immortalized epithelial cell lines, respectively. Cells were treated with drug and after 48–60 hours, percentage of living cells measured using Cell Titer Glo assay and normalized to untreated cells. Cancer cells can withstand higher doses (average LD_50_ = 2 µM), whereas epithelial cells are more sensitive to toxicity: lung (LD_50_ = 70 nM) and breast (average LD_50_ = 250 nM). Values are based off two experiments of six replicates.
(TIFF)

S3 Fig **CDDO-Me does not increase activation of Nrf2/ARE pathway in NSCLCs.** CDDO-Me does not affect expression of ARE-driven luciferase 18 hours after drug treatment in (A) A549, (B) H2009, (C) HCC 2429, and (D) HCC 4017. Firefly ARE-luciferase normalized to renilla control (RLU). Mean ± SEM of six replicates.
(TIFF)

S4 Fig **CDDO-Me protects** ***nrf2*** **-heterozygous but not** ***nrf2*** **-deficient mouse embryonic fibroblast (MEF) cells from 10 Gy radiation.** (A) Viable cells counts 48 hours post-IR show that 50 nM CDDO-Me increases the number of living *nrf2*+/− MEFs approximately 2-fold compared to cells treated with DMSO, whereas *nrf2*−/− MEFs are unprotected by CDDO-Me. (B) Total number of cells after IR. Mean ± SEM of triplicates.
(TIFF)

## References

[1] Ward JF (1988) DNA damage produced by ionizing radiation in mammalian cells: identities, mechanisms of formation, and reparability. Prog Nucleic Acid Res Mol Biol 35:95–125. 3065826 10.1016/s0079-6603(08)60611-x

[2] Bernier J, Hall EJ, Giaccia A (2004) Radiation oncology: a century of achievements. Nat Rev Cancer 4:737–747. 15343280 10.1038/nrc1451

[3] Cucinotta FA, Chappell LJ, Kim MH, Wang M (2012) Radiation carcinogenesis risk assessments for never-smokers. Health Phys 103:643–651. 23032894 10.1097/HP.0b013e318267b3ad

[4] Pearce MS, Salotti JA, Little MP, McHugh K, Lee C, et al (2012) Radiation exposure from CT scans in childhood and subsequent risk of leukaemia and brain tumours: a retrospective cohort study. Lancet 380:499–505. 22681860 10.1016/S0140-6736(12)60815-0 PMC3418594

[5] Newhauser WD, Durante M (2011) Assessing the risk of second malignancies after modern radiotherapy. Nat Rev Cancer 11:438–448. 21593785 10.1038/nrc3069 PMC4101897

[6] Ray PD, Huang BW, Tsuji Y (2012) Reactive oxygen species (ROS) homeostasis and redox regulation in cellular signaling. Cell Signal 24:981–990. 22286106 10.1016/j.cellsig.2012.01.008 PMC3454471

[7] Mitsuishi Y, Motohashi H, Yamamoto M (2012) The Keap1-Nrf2 system in cancers: stress response and anabolic metabolism. Front Oncol 2:200. 23272301 10.3389/fonc.2012.00200 PMC3530133

[8] Singh A, Misra V, Thimmulappa RK, Lee H, Ames S, et al (2006) Dysfunctional KEAP1-NRF2 interaction in non-small-cell lung cancer. PLoS Med 3:e420. 17020408 10.1371/journal.pmed.0030420 PMC1584412

[9] Solis LM, Behrens C, Dong W, Suraokar M, Ozburn NC, et al (2010) Nrf2 and Keap1 abnormalities in non-small cell lung carcinoma and association with clinicopathologic features. Clin Cancer Res 16:3743–3753. 20534738 10.1158/1078-0432.CCR-09-3352 PMC2920733

[10] Honda T, Yoshizawa H, Sundararajan C, David E, Lajoie MJ, et al (2011) Tricyclic compounds containing nonenolizable cyano enones. A novel class of highly potent anti-inflammatory and cytoprotective agents. J Med Chem 54:1762–1778. 21361338 10.1021/jm101445p PMC3251033

[11] Liby K, Royce DB, Williams CR, Risingsong R, Yore MM, et al (2007) The synthetic triterpenoids CDDO-methyl ester and CDDO-ethyl amide prevent lung cancer induced by vinyl carbamate in A/J mice. Cancer Res 67:2414–2419. 17363558 10.1158/0008-5472.CAN-06-4534

[12] Liby KT, Sporn MB (2012) Synthetic oleanane triterpenoids: multifunctional drugs with a broad range of applications for prevention and treatment of chronic disease. Pharmacol Rev 64:972–1003. 22966038 10.1124/pr.111.004846 PMC3462991

[13] Hong DS, Kurzrock R, Supko JG, He X, Naing A, et al (2012) A phase I first-in-human trial of bardoxolone methyl in patients with advanced solid tumors and lymphomas. Clin Cancer Res 18:3396–3406. 22634319 10.1158/1078-0432.CCR-11-2703 PMC4494099

[14] Liby K, Risingsong R, Royce DB, Williams CR, Yore MM, et al (2008) Prevention and treatment of experimental estrogen receptor-negative mammary carcinogenesis by the synthetic triterpenoid CDDO-methyl Ester and the rexinoid LG100268. Clin Cancer Res 14:4556–4563. 18628471 10.1158/1078-0432.CCR-08-0040 PMC5048101

[15] Liby K, Risingsong R, Royce DB, Williams CR, Ma T, et al (2009) Triterpenoids CDDO-methyl ester or CDDO-ethyl amide and rexinoids LG100268 or NRX194204 for prevention and treatment of lung cancer in mice. Cancer Prev Res (Phila) 2:1050–1058. 19952361 10.1158/1940-6207.CAPR-09-0085 PMC2818234

[16] Wang YY, Zhe H, Zhao R (2014) Preclinical evidences toward the use of triterpenoid CDDO-Me for solid cancer prevention and treatment. Mol Cancer 13:30. 24552536 10.1186/1476-4598-13-30 PMC3940295

[17] Liby K, Hock T, Yore MM, Suh N, Place AE, et al (2005) The synthetic triterpenoids, CDDO and CDDO-imidazolide, are potent inducers of heme oxygenase-1 and Nrf2/ARE signaling. Cancer Res 65:4789–4798. 15930299 10.1158/0008-5472.CAN-04-4539

[18] Liby KT, Royce DB, Risingsong R, Williams CR, Maitra A, et al (2010) Synthetic triterpenoids prolong survival in a transgenic mouse model of pancreatic cancer. Cancer Prev Res (Phila) 3:1427–1434. 20959520 10.1158/1940-6207.CAPR-10-0197 PMC2988079

[19] Daroczi B, Kari G, Ren Q, Dicker AP, Rodeck U (2009) Nuclear factor kappaB inhibitors alleviate and the proteasome inhibitor PS-341 exacerbates radiation toxicity in zebrafish embryos. Mol Cancer Ther 8:2625–2634. 19723885 10.1158/1535-7163.MCT-09-0198 PMC2846641

[20] Kim SB, Pandita RK, Eskiocak U, Ly P, Kaisani A, et al (2012) Targeting of Nrf2 induces DNA damage signaling and protects colonic epithelial cells from ionizing radiation. Proc Natl Acad Sci U S A 109:E2949–2955. 23045680 10.1073/pnas.1207718109 PMC3491493

[21] Eskiocak U, Kim SB, Roig AI, Kitten E, Batten K, et al (2010) CDDO-Me protects against space radiation-induced transformation of human colon epithelial cells. Radiat Res 174:27–36. 20681796 10.1667/RR2155.1

[22] Ramirez RD, Sheridan S, Girard L, Sato M, Kim Y, et al (2004) Immortalization of human bronchial epithelial cells in the absence of viral oncoproteins. Cancer Res 64:9027–9034. 15604268 10.1158/0008-5472.CAN-04-3703

[23] Sato M, Vaughan MB, Girard L, Peyton M, Lee W, et al (2006) Multiple oncogenic changes (K-RAS(V12), p53 knockdown, mutant EGFRs, p16 bypass, telomerase) are not sufficient to confer a full malignant phenotype on human bronchial epithelial cells. Cancer Res 66:2116–2128. 16489012 10.1158/0008-5472.CAN-05-2521

[24] Shay JW, Tomlinson G, Piatyszek MA, Gollahon LS (1995) Spontaneous in vitro immortalization of breast epithelial cells from a patient with Li-Fraumeni syndrome. Mol Cell Biol 15:425–432. 7799951 10.1128/mcb.15.1.425 PMC231985

[25] Shay JW, Van Der Haegen BA, Ying Y, Wright WE (1993) The frequency of immortalization of human fibroblasts and mammary epithelial cells transfected with SV40 large T-antigen. Exp Cell Res 209:45–52. 8224005 10.1006/excr.1993.1283

[26] Ramos-Gomez M, Kwak MK, Dolan PM, Itoh K, Yamamoto M, et al (2001) Sensitivity to carcinogenesis is increased and chemoprotective efficacy of enzyme inducers is lost in nrf2 transcription factor-deficient mice. Proc Natl Acad Sci U S A 98:3410–3415. 11248092 10.1073/pnas.051618798 PMC30667

[27] Pike MC, Alper T (1964) A Method for Determining Dose-Modification Factors. Br J Radiol 37:458–462. 14168493 10.1259/0007-1285-37-438-458

[28] Gyori BM, Venkatachalam G, Thiagarajan PS, Hsu D, Clement MV (2014) OpenComet: An automated tool for comet assay image analysis. Redox Biol 2:457–465. 24624335 10.1016/j.redox.2013.12.020 PMC3949099

[29] Casas F, Vinolas N (2004) Toxicity of small cell lung cancer treatment. Hematol Oncol Clin North Am 18:461–481. 15094182 10.1016/j.hoc.2003.12.007

[30] Andreassen CN, Grau C, Lindegaard JC (2003) Chemical radioprotection: a critical review of amifostine as a cytoprotector in radiotherapy. Semin Radiat Oncol 13:62–72. 12520465 10.1053/srao.2003.50006

[31] Wasserman TH, Brizel DM (2001) The role of amifostine as a radioprotector. Oncology (Williston Park) 15:1349–1354 discussion 1357–1360. 11702962

[32] Radford EP (1980) Human health effects of low doses of ionizing radiation: the BEIR III controversy. Radiat Res 84:369–394. 6893866

[33] Meadows AT, Gallagher JA, Bunin GR (1992) Late effects of early childhood cancer therapy. Br J Cancer Suppl 18 S92–95. PMC2149648 1503934

[34] Kim YH, Yoo KC, Cui YH, Uddin N, Lim EJ, et al (2014) Radiation promotes malignant progression of glioma cells through HIF-1alpha stabilization. Cancer Lett 354:132–141. 25109450 10.1016/j.canlet.2014.07.048

[35] Koukourakis MI, Giatromanolaki A, Chong W, Simopoulos C, Polychronidis A, et al (2004) Amifostine induces anaerobic metabolism and hypoxia-inducible factor 1 alpha. Cancer Chemother Pharmacol 53:8–14. 14574457 10.1007/s00280-003-0691-z

[36] Levy S, Forman HJ (2010) C-Myc is a Nrf2-interacting protein that negatively regulates phase II genes through their electrophile responsive elements. IUBMB Life 62:237–246. 20232342 10.1002/iub.314 PMC2852429

[37] Shanmugam MK, Dai X, Kumar AP, Tan BK, Sethi G, et al (2014) Oleanolic acid and its synthetic derivatives for the prevention and therapy of cancer: preclinical and clinical evidence. Cancer Lett 346:206–216. 24486850 10.1016/j.canlet.2014.01.016 PMC4004441

[38] Pergola PE, Raskin P, Toto RD, Meyer CJ, Huff JW, et al (2011) Bardoxolone methyl and kidney function in CKD with type 2 diabetes. N Engl J Med 365:327–336. 21699484 10.1056/NEJMoa1105351

[39] Liby KT (2014) Synthetic triterpenoids can protect against toxicity without reducing the efficacy of treatment with Carboplatin and Paclitaxel in experimental lung cancer. Dose Response 12:136–151. 24659938 10.2203/dose-response.13-018.Liby PMC3960959

