# Supplementary figures and images for: CDDO-Me Protects Normal Lung and Breast Epithelial Cells but Not Cancer Cells from Radiation
Source: PLoS One. 2014 Dec 23;9(12):e115600. doi: 10.1371/journal.pone.0115600 (PMC4275221; doi:10.1371/journal.pone.0115600)

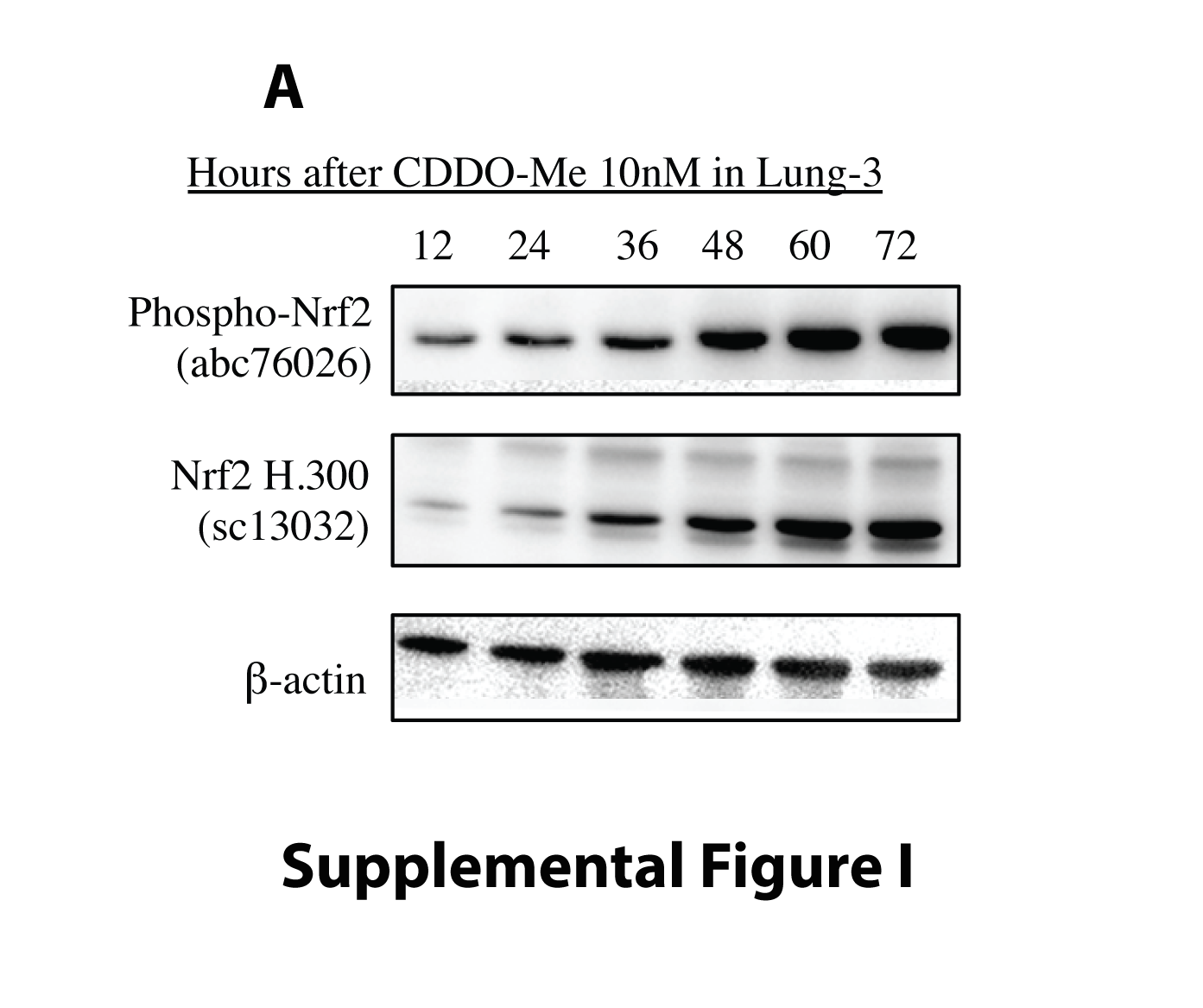

Supplement: S1 Fig — CDDO-Me increases Nrf2 protein over time. (A) Protein levels of phosphor-Nrf2 (band observed at ∼120 kDa) and total Nrf2 (bands observed at ∼68, 75 kDa) after treatment with 10 nM CDDO-Me in HBEC 3KT. (TIFF) [file pone.0115600.s001.tiff]

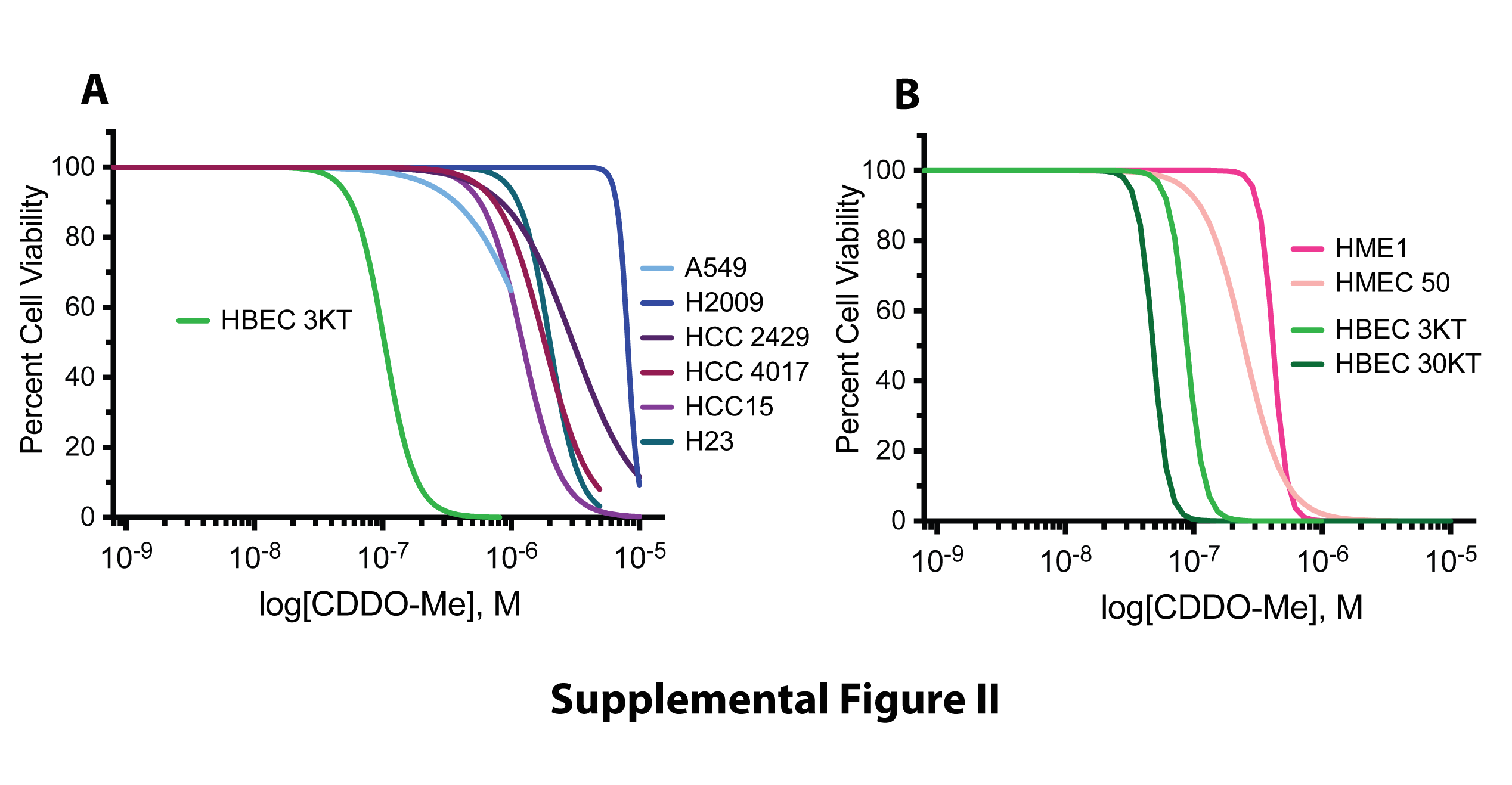

Supplement: S2 Fig — Epithelial cells are more sensitive to CDDO-Me when compared to cancer cells. Cell Titer Glo toxicity curves of various (A) NSCLCs and (B) immortalized epithelial cell lines, respectively. Cells were treated with drug and after 48–60 hours, percentage of living cells measured using Cell Titer Glo assay and normalized to untreated cells. Cancer cells can withstand higher doses (average LD50 = 2 µM), whereas epithelial cells are more sensitive to toxicity: lung (LD50 = 70 nM) and breast (average LD50 = 250 nM). Values are based off two experiments of six replicates. (TIFF) [file pone.0115600.s002.tiff]

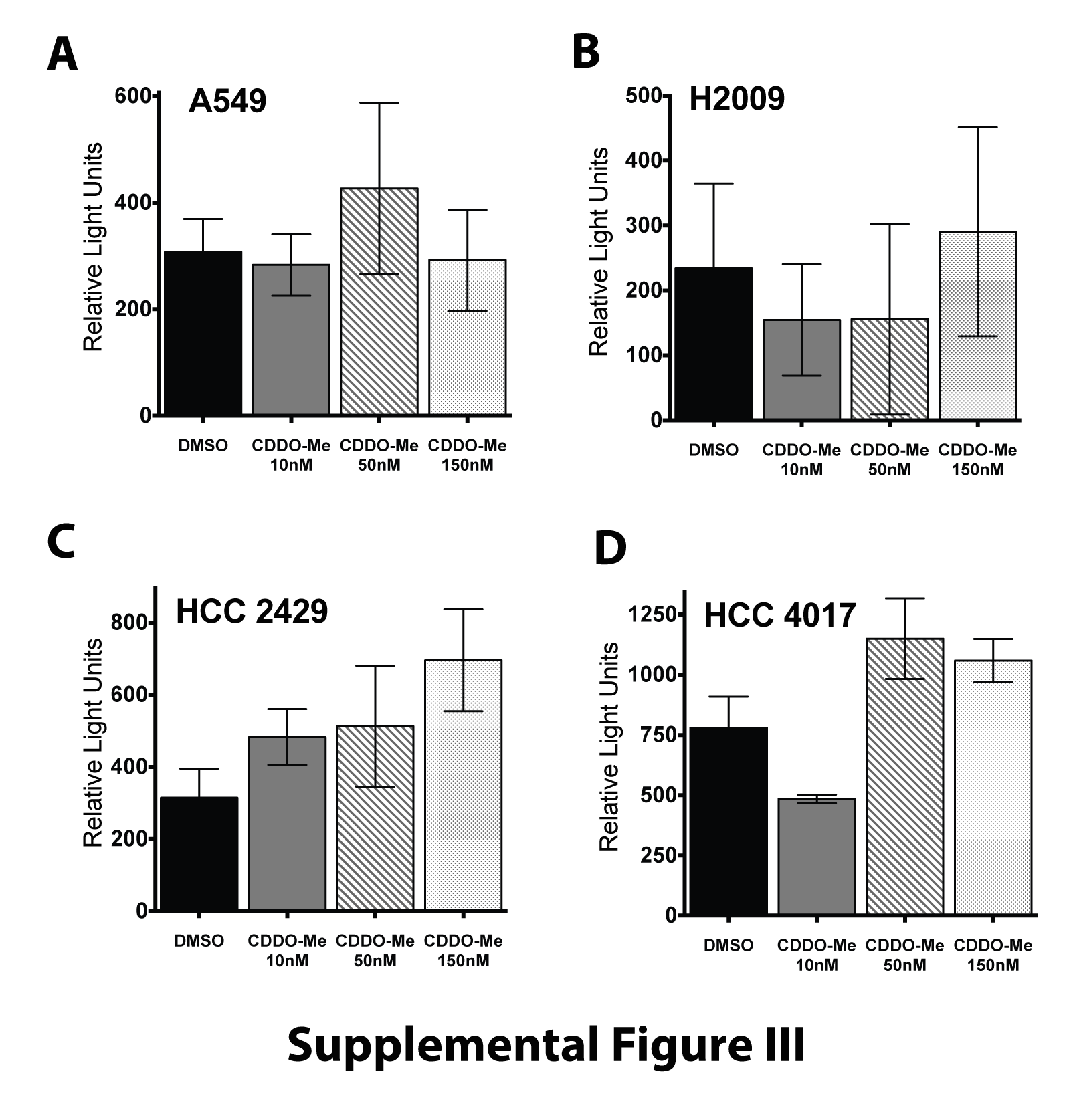

Supplement: S3 Fig — CDDO-Me does not increase activation of Nrf2/ARE pathway in NSCLCs. CDDO-Me does not affect expression of ARE-driven luciferase 18 hours after drug treatment in (A) A549, (B) H2009, (C) HCC 2429, and (D) HCC 4017. Firefly ARE-luciferase normalized to renilla control (RLU). Mean ± SEM of six replicates. (TIFF) [file pone.0115600.s003.tiff]

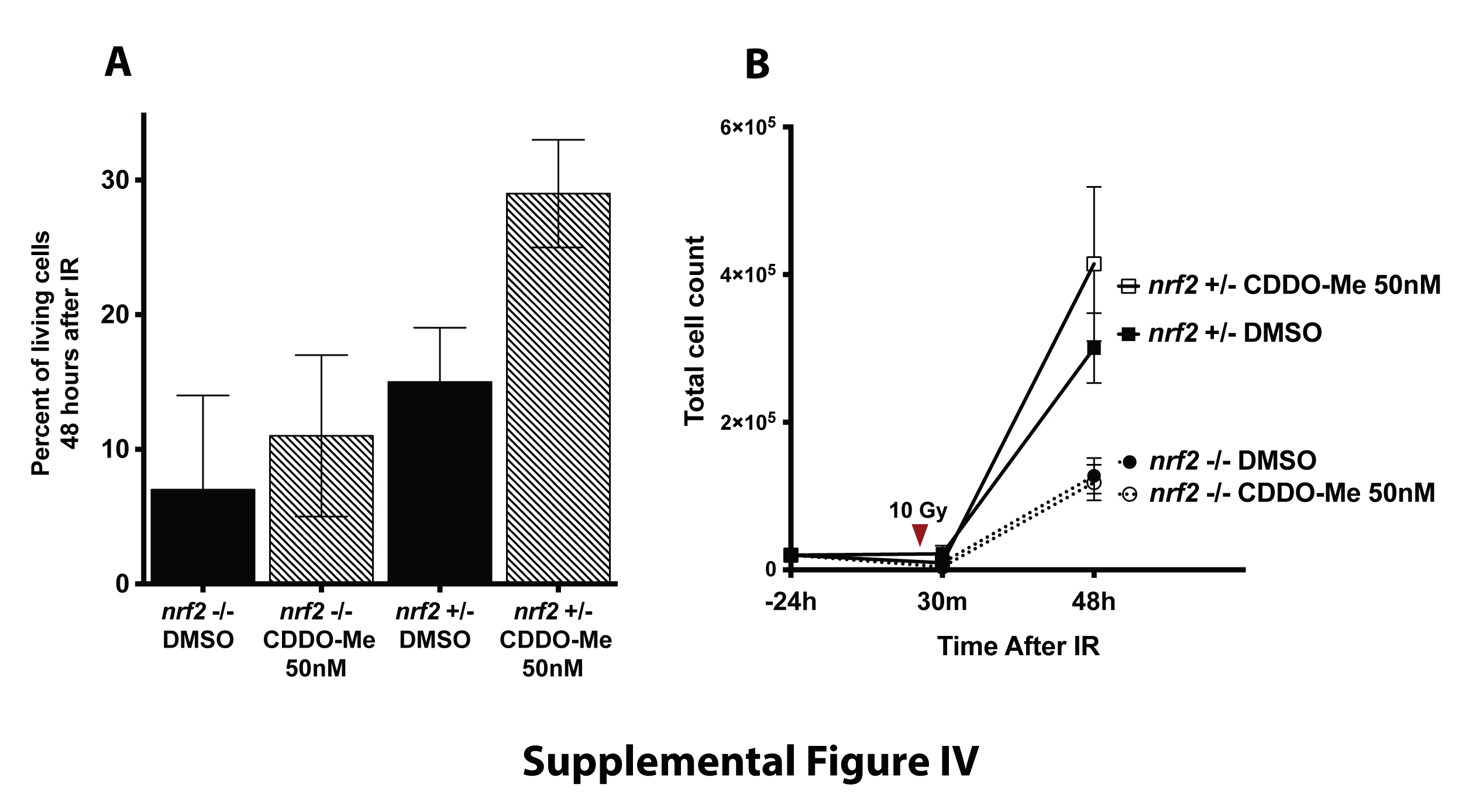

Supplement: S4 Fig — CDDO-Me protects nrf2 -heterozygous but not nrf2 -deficient mouse embryonic fibroblast (MEF) cells from 10 Gy radiation. (A) Viable cells counts 48 hours post-IR show that 50 nM CDDO-Me increases the number of living nrf2+/− MEFs approximately 2-fold compared to cells treated with DMSO, whereas nrf2−/− MEFs are unprotected by CDDO-Me. (B) Total number of cells after IR. Mean ± SEM of triplicates. (TIFF) [file pone.0115600.s004.tiff]
